# Analysis of key genes and their functions in placental tissue of patients with gestational diabetes mellitus

**DOI:** 10.1186/s12958-019-0546-z

**Published:** 2019-11-29

**Authors:** Yuxia Wang, Haifeng Yu, Fangmei Liu, Xiue Song

**Affiliations:** 1 Department of Gynecology, Jinan Central Hospital, Jinan City, 250013 Shandong Province China; 2 Department of Obstetrics, Jinan Central Hospital, No. 105 Jiefang Road, Lixia District, Jinan City, 250013 Shandong Province China

**Keywords:** Gestational diabetes mellitus, Differentially expressed genes, Protein-protein interaction network, Integrated regulatory network, Transcription factors

## Abstract

**Background:**

This study was aimed at screening out the potential key genes and pathways associated with gestational diabetes mellitus (GDM).

**Methods:**

The GSE70493 dataset used for this study was obtained from the Gene Expression Omnibus database. Differentially expressed genes (DEGs) in the placental tissue of women with GDM in relation to the control tissue samples were identified and submitted to protein-protein interaction (PPI) network analysis and subnetwork module mining. Functional enrichment analyses of the PPI network and subnetworks were subsequently carried out. Finally, the integrated miRNA–transcription factor (TF)–DEG regulatory network was analyzed.

**Results:**

In total, 238 DEGs were identified, of which 162 were upregulated and 76 were downregulated. Through PPI network construction, 108 nodes and 278 gene pairs were obtained, from which chemokine (C-X-C motif) ligand 9 (*CXCL9*), *CXCL10*, protein tyrosine phosphatase, receptor type C (*PTPRC*), and human leukocyte antigen (*HLA*) were screened out as hub genes. Moreover, genes associated with the immune-related pathway and immune responses were found to be significantly enriched in the process of GDM. Finally, miRNAs and TFs that target the DEGs were predicted.

**Conclusions:**

Four candidate genes (viz., *CXCL9*, *CXCL10*, *PTPRC*, and *HLA*) are closely related to GDM. *miR-223-3p*, *miR-520*, and thioredoxin-binding protein may play important roles in the pathogenesis of this disease.

## Background

Expectant mothers with gestational diabetes mellitus (GDM), a common pregnancy complication, have an increased risk of developing type 2 diabetes mellitus [1]. Over the past 20 years, the prevalence of GDM has doubled, affecting approximately 10% of pregnancies in the USA [2, 3]. Babies born to mothers with GDM are typically at a high risk for macrosomia, neonatal cardiac dysfunction, neonatal hypoglycemia, stillbirth, childhood obesity, and type 2 diabetes mellitus [4–6]. Given the worldwide prevalence and adverse outcomes of GDM, there is an urgent need to grasp the pathophysiology and pathogenesis of the disease [2].

Previous studies have suggested that GDM is caused by enhanced insulin resistance and pancreatic beta (β)-cell dysfunction [7], involving genes that are related to insulin signaling, insulin secretion, maturity-onset diabetes of the young, and lipid and glucose metabolism, to name a few [8, 9]. Subsequently, it was found that inflammatory pathways [10], metabolic disorder [11], oxidative stress [12], and vitamin D concentrations [13] were also related to GDM. Furthermore, some genetic alterations, such as those of the genes encoding β3-adrenergic receptor [14] and transcription factor 7-like 2 polymorphism [15], were also found to be associated with GDM. Moreover, GDM results in major changes in the expression profiles of placental genes, with a significant increase in markers and mediators of inflammation [10]. Recently, several microarray studies have verified that the cytochrome P450, family 1, subfamily A, polypeptide 1 (*CYP1A1*), estrogen receptor 1 (*ESR1*) [16], fibronectin 1 (*FN1*), and leptin (*LEP*) [17] genes were essential for the pathogenesis of GDM. However, because the genes related to GDM have not yet been fully identified, the biological processes underlying the pathogenesis of this disease remain unclear.

In this study, the gene expression profiles of placental tissue from women with GDM were compared with those of matched normal placental tissue by microarray analysis, to screen out differentially expressed genes (DEGs) in GDM. The identified DEGs were then submitted to Kyoto Encyclopedia of Genes and Genomes (KEGG) pathway and Gene Ontology (GO) enrichment analyses to explore the crucial pathways of GDM. Additionally, a protein-protein interaction (PPI) network was constructed and subnetwork module mining was performed to seek out the candidate disease genes. Finally, microRNAs (miRNAs) and transcription factors (TFs) that target the candidate DEGs were identified and analyzed. The results from this study may lay the groundwork for future research on the pathogenesis of GDM.

## Methods

### Microarray analysis

The gene expression dataset GSE70493, which is based on the GPL17586 [HTA-2_0] Affymetrix Human Transcriptome Array 2.0 [transcript (gene) version] platform, was downloaded from the National Center for Biotechnology Information’s Gene Expression Omnibus database (http://www.ncbi.nlm.nih.gov/geo/). This dataset comprised 63 placental tissue specimens collected from 32 cases of GDM and 31 matched pregnancies without maternal complications.

### Data reprocessing

The probe-level data (CEL files) were converted to expression estimates by the Puma [18] and Oligo [19] packages in R, and the original expression dataset was processed into expression values using the robust multi-array average algorithm [20] with the default settings implemented in Bioconductor. The DEGs were identified with the limma [21] software package according to the expression values of the sample probes, and only those with a *p*-value of less than 0.01 were selected and annotated for further analysis.

### GO and KEGG pathway enrichment analyses

To assess the functions and significantly enriched pathways of the DEGs, ClusterProfiler [22] was used to identify the overrepresented GO terms in the biological process (BP), cellular component (CC), and molecular function (MF) categories, as well as the KEGG pathway categories. The hypergeometric distribution threshold for these analyses was a *p*-value of < 0.05.

### PPI network construction and subnetwork module mining

The Search Tool for the Retrieval of Interacting Genes (STRING, ver. 10.0, https://string-db.org/) [23] database was used to analyze functional interactions between the DEGs and other genes, under a confidence score threshold of > 0.4. The PPI network was established using Cytoscape (ver. 3.3.0, http://www.cytoscape.org/) [24]. Then, the topology of the network was analyzed, and the hub nodes in the network were obtained by calculating the average degree of each node. The average degree is the average number of edges connecting all the nodes in the network, measured by three indexes: degree centrality [25], betweenness centrality [26], and closeness centrality [27].

Molecular Complex Detection (MCODE) [28] is an automated method for searching molecular complexes with similar functions in large protein interaction networks. The MCODE (ver. 1.4.2, http://apps.cytoscape.org/apps/mcode) plugin of Cytoscape was used to analyze the subnetwork modules with similar functions in the original PPI network. Then, GO and KEGG pathway analyses of the subnetwork modules were performed to evaluate their functions.

### Prediction of miRNAs and transcription factors that regulate the DEGs

The TFs associated with the DEGs were predicted by the position weight matrices from TRANSFAC and JASPAR in the Enrichr database [29], under the hypergeometric distribution threshold of *p* < 0.01. miRNAs associated with the DEGs were predicted by miRTarBase in the Enrichr database, under the hypergeometric distribution threshold of *p* < 0.01. On the basis of the miRNA–DEG regulatory network and TF–DEG regulatory network, the integrated DEG–miRNA–TF regulatory network was constructed using Cytoscape.

## Results

### Analysis of the differentially expressed genes

**Fig. 1:**
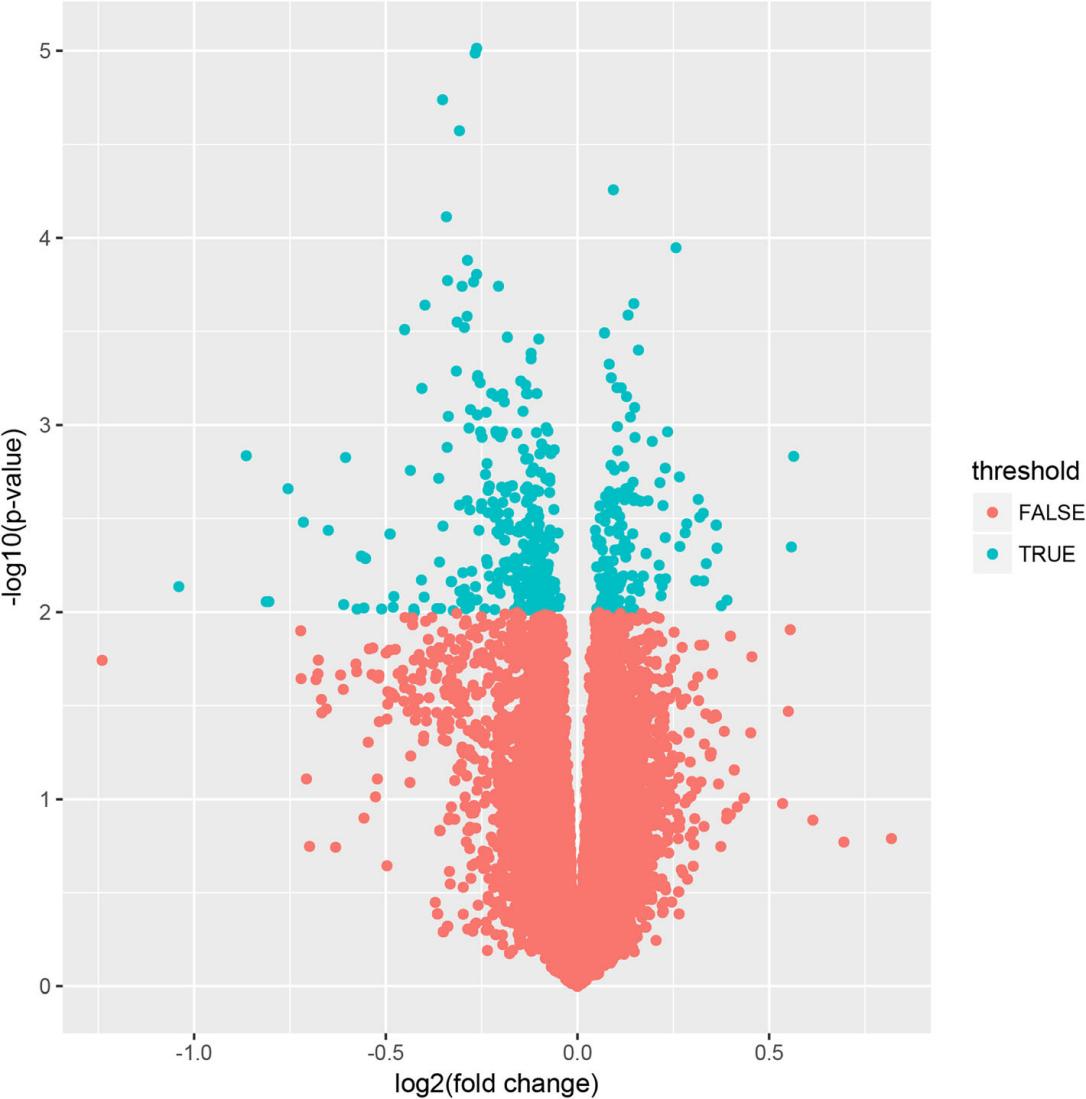
Volcano map of the distribution of differentially expressed genes. Each blue dot represents a differentially expressed gene

After the microarray analysis, the probes that were mapped to multiple genes were considered nonspecific and were removed, and only those with unique genes were distinguished as DEGs. In total, 238 DEGs (comprising 162 upregulated and 76 downregulated genes) were identified from the GDM placental tissue samples compared with the matching normal pregnant tissue samples (Fig. 1).

### Functional enrichment analyses

**Fig. 2:**
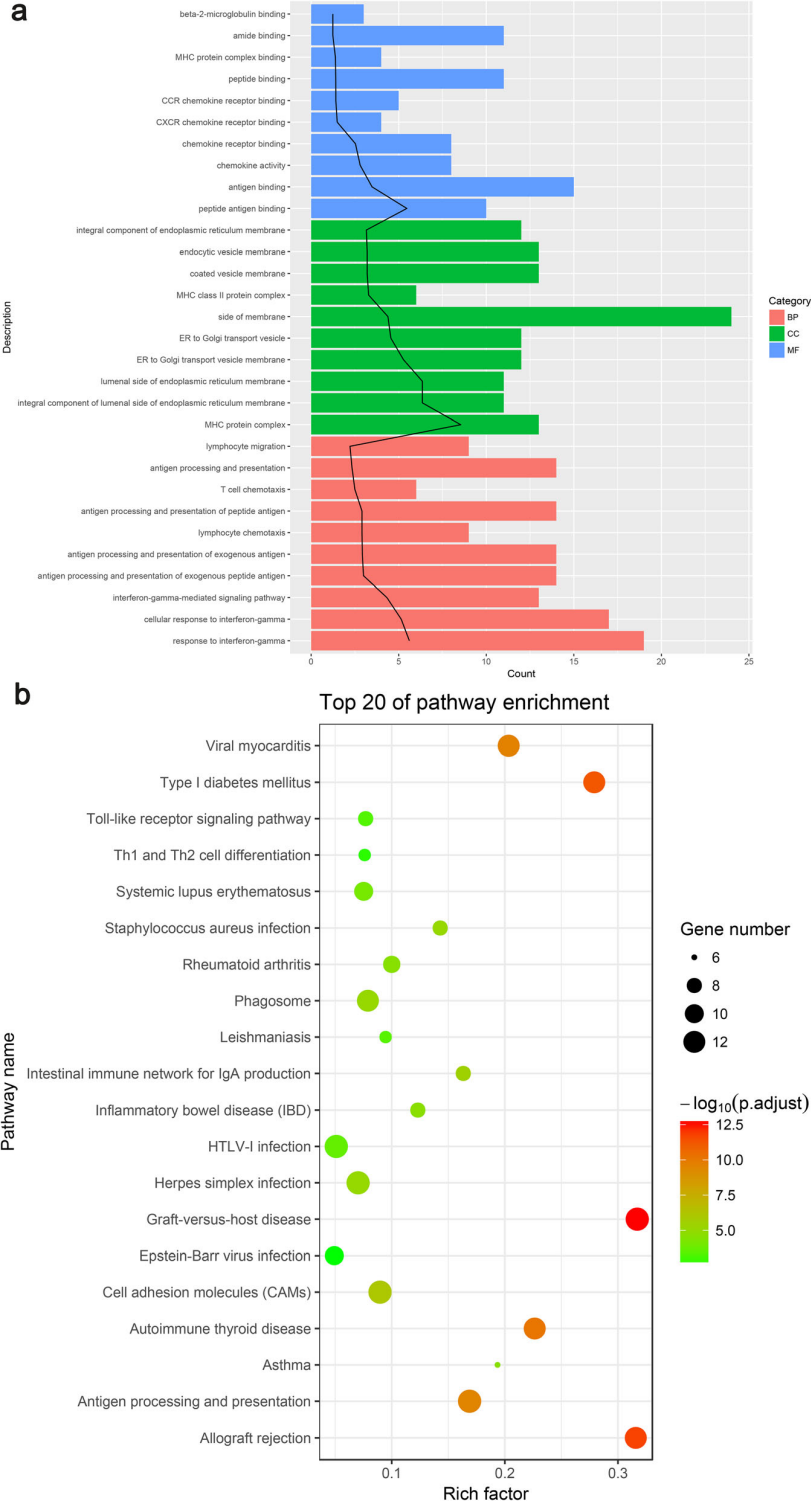
GO and KEGG pathway enrichment analyses of the differentially expressed genes. **a** Gene Ontology (GO) enrichment analysis of the top 10 differentially expressed genes (DEGs) by *p*-value. BP: Biological process; MF: molecular function; CC: cellular component; Counts: the number of enriched DEGs; Black trend line: -log_10_ (p-adjust)/2; P-adjust: rectified *p*-value. **b** Kyoto Encyclopedia of Genes and Genomes (KEGG) pathway enrichment analysis of the DEGs. Rich factor: the ratio of the number of enriched DEGs in the KEGG category to the total genes in that category. The larger the Rich factor, the higher the degree of enrichment

Through GO analysis, the top 10 overrepresented GO terms in the BP, MF, and CC categories were identified on the basis of the *p*-value (Fig. 2a). In the BP category, the overrepresented terms included the interferon-gamma-mediated signaling pathway, lymphocyte chemotaxis, antigen processing and presentation of exogenous peptide antigen, and lymphocyte chemotaxis. In the CC category, the major histocompatibility complex (MHC) proteins, endoplasmic reticulum membrane, coated vesicle membrane, and endocytic vesicle membrane terms were enriched. In the MF category, the most significantly enriched terms were antigen binding, chemokine receptor binding, and MHC protein complex binding. The most remarkable DEGs involved in those GO terms encoded the human leukocyte antigen (*HLA*), chemokine (C-X-C motif) ligand 9 (*CXCL9)*, *CXCL10*, chemokines (*CCL3*, *CCL4L1*, *CCL4*, and *CCL8*), and protein tyrosine phosphatase, receptor type C (*PTPRC*).

Through KEGG pathway enrichment analysis, the top 20 enriched pathways were identified on the basis of the *p*-value (Fig. 2b). The significantly enriched categories included pathways for allograft rejection, graft-versus-host disease, type I diabetes mellitus, autoimmune thyroid disease, inflammatory bowel disease, the Toll-like receptor signaling pathway, and Th1 and Th2 cell differentiation. The genes associated with those pathways were *HLA*, *CXCL9*, *CXCL10*, and *PTPRC*.

### PPI network and subnetwork module analyses

**Fig. 3:**
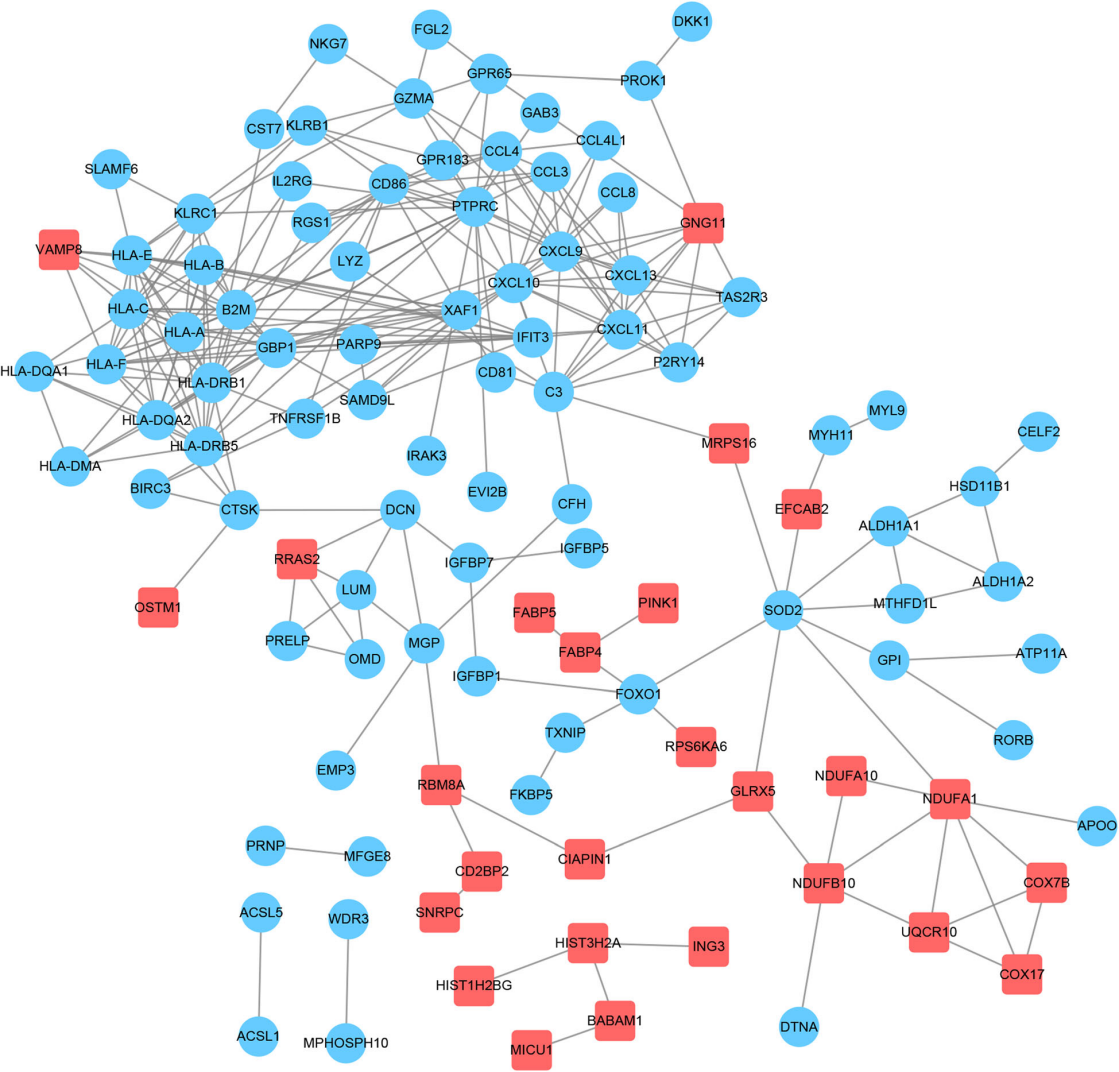
Protein-protein interaction network of the differentially expressed genes. The red square node represents upregulated genes; the blue circular node represents downregulated genes

From the PPI network analysis, 108 nodes and 278 gene pairs were acquired (Fig. 3) and the top 15 nodes according to the measured scores of the three centrality indexes were screened out (Table 1). Among these, *PTPRC*, *CXCL9*, and *CXCL10* always ranked in the top 15 for each index, implying that these genes may play important roles in the progression of GDM.

**Table 1:** Node genes measured in three indexes (top 15)

Gene	Degree	Gene	Betweenness	Gene	Closeness
*PTPRC*	22	*SOD2*	3911.9185	*C3*	0.072248
*CXCL10*	18	*C3*	3533.4678	*CXCL10*	0.071716
*HLA-A*	18	*MRPS16*	2901.8518	*CXCL9*	0.07162
*HLA-DRB1*	17	*PTPRC*	1506.8242	*CXCL11*	0.071096
*GBP1*	16	*FOXO1*	1442.8683	*CXCL13*	0.071096
*CXCL9*	15	*DCN*	1370.2727	*PTPRC*	0.07072
*HLA-C*	15	*CTSK*	1234.5901	*MRPS16*	0.070674
*B2M*	15	*CXCL10*	1129.5261	*LYZ*	0.070627
*HLA-E*	15	*NDUFA1*	1000.009	*CD81*	0.070256
*HLA-DQA2*	13	*MGP*	953.21423	*GNG11*	0.07021
*HLA-F*	13	*CXCL9*	760.1234	*GBP1*	0.070072
*HLA-DRB5*	13	*HLA-DRB1*	755.0963	*CD86*	0.070072
*HLA-B*	13	*IGFBP7*	708.1684	*B2M*	0.070026
*CD86*	12	*RBM8A*	625.97986	*TAS2R3*	0.06998
*CXCL11*	12	*IGFBP1*	538.0367	*P2RY14*	0.06998

**Fig. 4:**
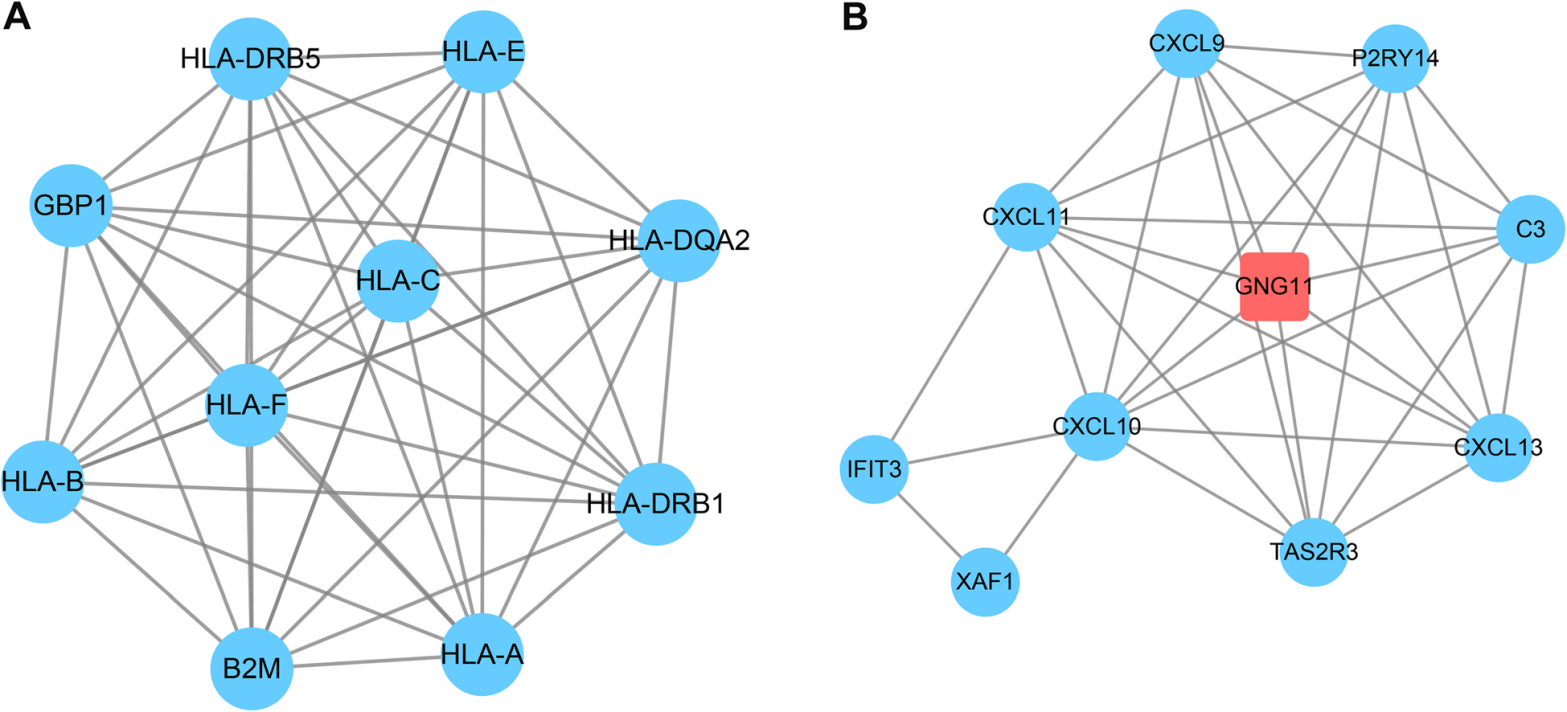
Two subnetwork modules of the differentially expressed genes. **a** Module 1 subnetwork diagram; **b** module 2 subnetwork diagram. The red square nodes represent upregulated genes; the blue round nodes represent downregulated genes

**Fig. 5:**
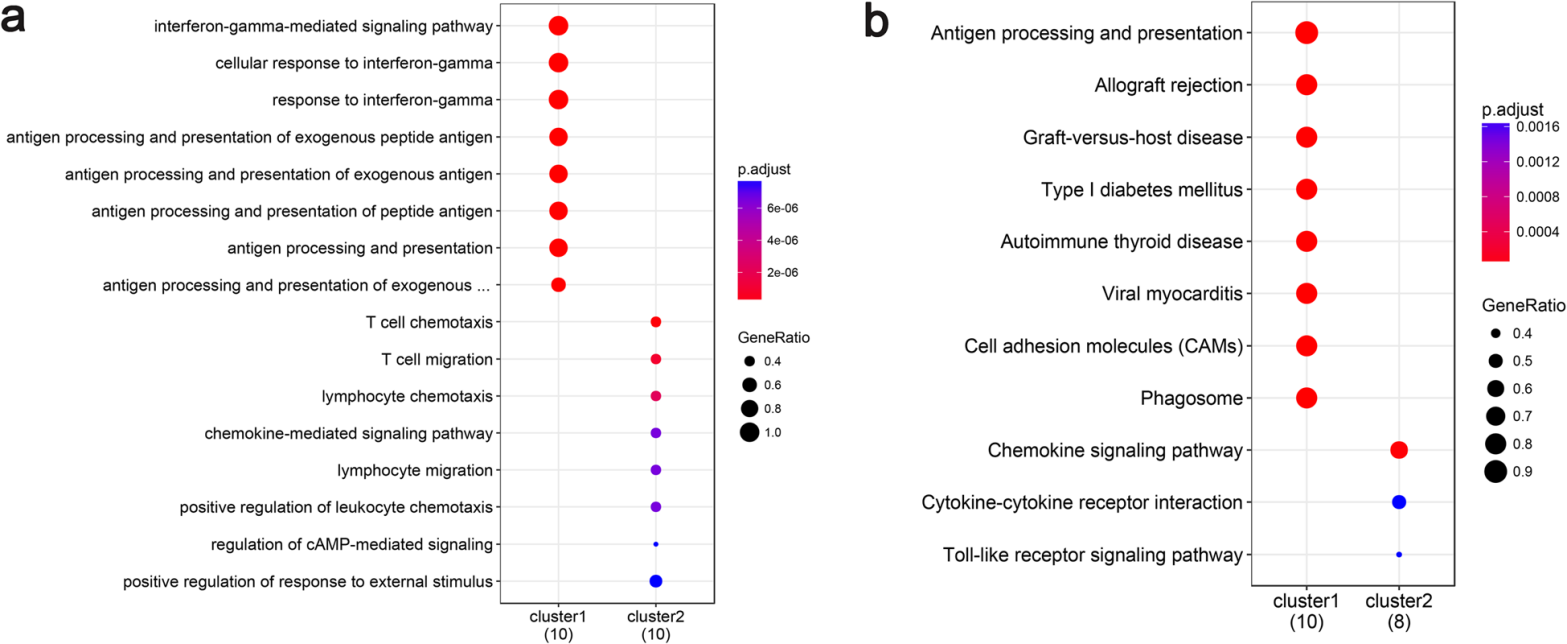
GO and KEGG pathway enrichment analyses of the differentially expressed genes in two subnetwork modules. **a** Gene Ontology (GO) enrichment analysis of the differentially expressed genes (DEGs) in the two subnetwork modules. GeneRatio: the ratio of the number of lncRNA target genes in the GO category to that of the annotated genes (counts below the horizontal axis) in the GO database. The horizontal coordinate is the lncRNA, and the ordinate is the name of the GO category. **b** Kyoto Encyclopedia of Genes and Genomes (KEGG) pathway enrichment analysis of the DEGs in the two subnetwork modules. GeneRatio: the ratio of the number of lncRNA target genes in the KEGG category to that of the annotated genes (counts below the horizontal axis) in the KEGG database. The horizontal coordinate is the lncRNA, and the ordinate is the name of the KEGG item

Two significant modules with scores > 5 and nodes > 5 were isolated from the PPI network (Fig. 4). *HLA* was found to be spread all over module 1. Functional analysis of the DEGs in module 1 verified that the GO terms were strongly related to antigen processing presentation and autoimmune thyroid disease (Fig. 5a). In module 2, *CXCL9* and *CXCL10* with the higher degrees were involved in lymphocyte chemotaxis and the chemokine signaling pathway (Fig. 5b).

### Prediction of miRNAs and transcription factors that regulate the target genes

**Fig. 6:**
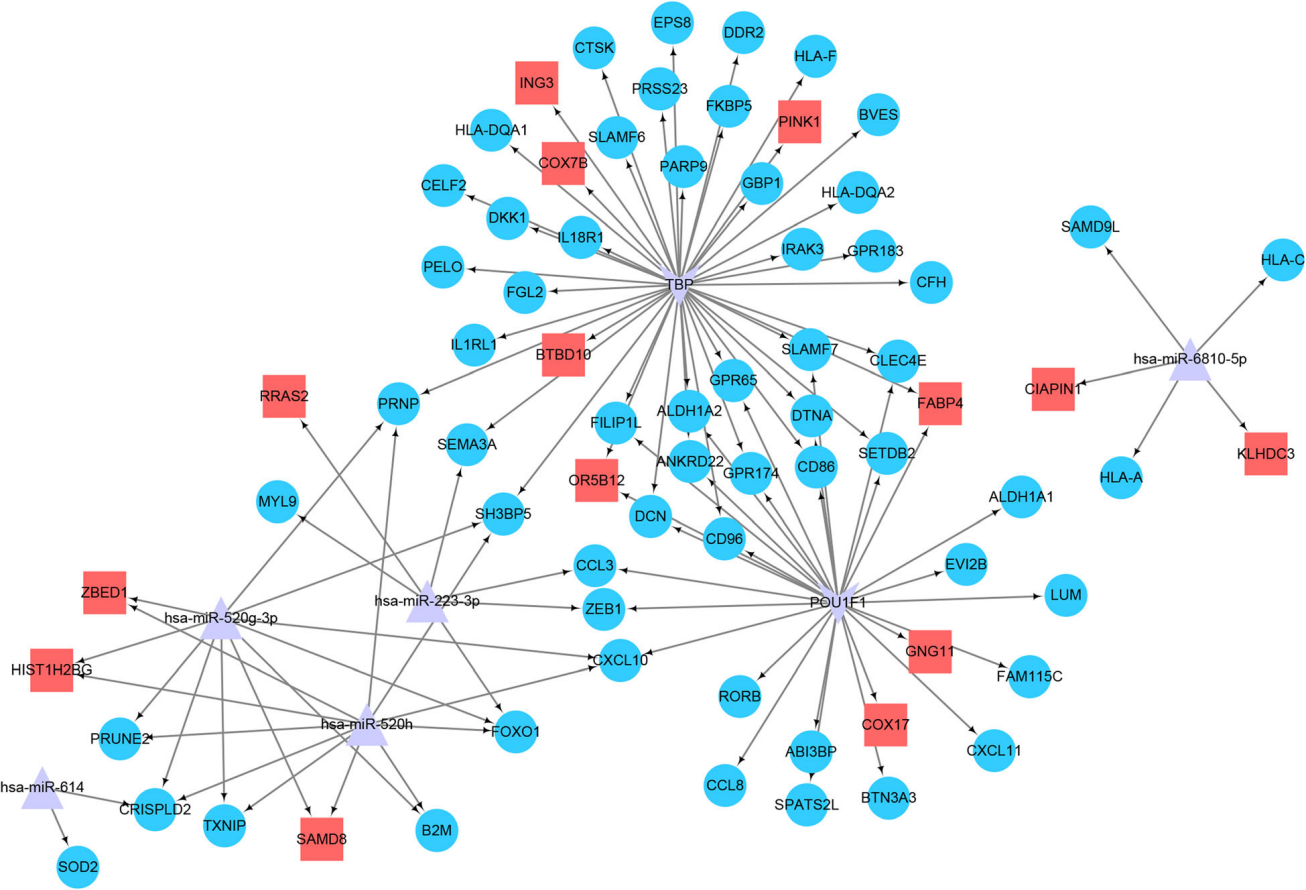
Constructed interaction network of the differentially expressed genes. The red square nodes are upregulated genes; the blue circle nodes are downregulated genes; the purple v-shaped frames are transcription factors (TFs); and the purple triangles are miRNAs

The miRNAs and TFs that may regulate the target gene were obtained from the Enrichr database [29] (Table 2). The miRNA–DEG–TF regulatory network was constructed by integrating the DEGs with the DEG-related miRNAs and TFs, as shown in Fig. 6. The integrated network comprised five miRNAs, 73 DEGs (59 down-regulated and 14 up-regulated), and two TFs (thioredoxin-binding protein (TBP) and POU class 1 homeobox 1 (POU1F1)). Specifically, most of the DEGs were regulated by *miR-223-3p*, *miR-520*, and TBP.

**Table 2:** miRNAs and transcription factors (TFs) regulating DEGs

Term	Gene count	P-value	Genes
POU1F1	29	0.0027516	*CD86;GPR65;COX17;RORB;GPR174;CCL8;ABI3BP;CCL3;* *SLAMF7;OR5B12;FILIP1L;CD96;DTNA;LUM;ANKRD22;* *SETDB2;BTN3A3;GNG11;DCN;CXCL10;CXCL11;ZEB1;* *FAM115C;FABP4;ALDH1A2;ALDH1A1;SPATS2L;EVI2B;* *CLEC4E*
TBP	43	0.0072984	*CD86;COX7B;CFH;CELF2;GPR65;SEMA3A;FGL2;C2ORF88;* *PRSS23;EPS8;GPR174;IL1RL1;ING3;CTSK;SH3BP5;SLAMF7;* *OR5B12;SLAMF6;FILIP1L;BVES;PELO;GBP1;HLA-DQA2;* *HLA-DQA1;PRNP;CD96;DTNA;ANKRD22;SETDB2;IRAK3;* *HLA-F;PARP9;BTBD10;DKK1;DCN;PINK1;FABP4;GPR183;* *ALDH1A2;CLEC4E;IL18R1;FKBP5;DDR2*
hsa-miR-223-3p	6	0.0011276	*ZEB1;SEMA3A;CCL3;RRAS2;MYL9;FOXO1*
hsa-miR-614	2	0.0048044	*CRISPLD2;SOD2*
hsa-miR-6810-5p	5	0.0087679	*KLHDC3;SAMD9L;HLA-C;CIAPIN1;HLA-A*
hsa-miR-520 g-3p	11	0.0093275	*CXCL10;PRNP;SAMD8;CRISPLD2;PRUNE2;SH3BP5;TXNIP;* *ZBED1;HIST1H2BG;B2M;FOXO1*
hsa-miR-520 h	11	0.0098226	*CXCL10;PRNP;SAMD8;CRISPLD2;PRUNE2;SH3BP5;TXNIP;* *ZBED1;HIST1H2BG;B2M;FOXO1*

## Discussion

GDM describes the condition of abnormal sugar metabolism or potential decreased glucose tolerance before pregnancy and is confirmed during pregnancy [30–32]. It is a complex disease, being influenced by many factors such as the environment, society, and genes [33]. Moreover, genetic studies have suggested that multiple genes are involved in the disease [8]. In our study, DEGs in GDM and their enriched functions were screened out via bioinformatic analysis, and four key genes (viz., *HLA*, *CXCL9*, *CXCL10*, and *PTPRC*) were identified to be crucial to the disease. Moreover, *miR-223-3p*, *miR-520*, and TBP were found to be strongly linked to those DEGs, indicating their importance in GDM.

*CXCL9* and *CXCL10* are categorized as “inflammatory” chemokines. Shimada and coworkers postulated that the binding of *CXCL10* to *CXCR3* played a crucial role in the suppression of pancreatic β-cell proliferation [34]. Besides this, *CXCL10* could interact with Toll-like receptor 4 to continuously activate c-Jun N-terminal kinases and protein kinase B (Akt), induce the cleavage of p21-activated protein kinase 2, and switch the Akt signal from proliferation to apoptosis, resulting in the suppression of pancreatic β-cell proliferation [35]. The present study demonstrated that *CXCL10* was significantly enriched in the Toll-like receptor signaling pathway, leading us to speculate that it is a key gene that participates in the pathogenesis of GDM by regulating the progress of the Toll-like receptor signaling pathway. Although *CXCL9* has similar functional and structural characteristics as *CXCL10*, it was reported that *CXCL9* could not bind to Toll-like receptor 4 [36]. In this study, *CXCL9* was significantly enriched in the cytokine signaling pathway and may thus play a critical role in the pathogenesis of GDM by regulating the inflammatory pathway.

*HLA*, the gene for the human MHC, plays a pivotal role in the antigen presentation of extracellular and intracellular peptides and the regulation of immune responses [37]. Compared with other regions of the human genome, the MHC genes on chromosome 6 are more associated with the susceptibility to common diseases like diabetes, and indeed many reports have shown that *HLA* gene variants are related to the predisposition to type 1 diabetes mellitus [38]. Additionally, although type 2 diabetes mellitus is not an autoimmune disease or associated with the *HLA* gene, there is evidence that genes in the *HLA* region might have an influence on the genetic susceptibility to this metabolic disorder [39]. Importantly, Steinborn and colleagues found that GDM was related to an increased humoral immune response against *HLA*-class II antigens [40]. Our study highlights the importance of *HLA* in the progression of GDM, during which the gene is downregulated, and emphasizes that the autoimmune response is significantly associated with the disease pathogenesis.

*PTPRC* (*CD45*) has an essential role in lymphocyte development, antigen receptor signal transduction, and modulation of the signals emanating from integrin and cytokine receptors [41]. In diabetes mellitus, protein tyrosine phosphatases act as negative regulators of insulin signal transduction [42]. A previous study demonstrated that the homozygous deletion of protein tyrosine phosphatase 1B (*PTP1B*) in myocytes enhanced both the insulin-dependent activation of insulin receptor autophosphorylation and the tyrosine phosphorylation of insulin receptor substrates, and increased insulin sensitivity [43]. Moreover, it was shown that the expression of *PTPRC* was related to residual β-cell function in type 1 diabetes mellitus [44]. Our results reveal that *PTPRC* is likely to be a key gene that impacts GDM.

Because *miR-223* was found to be significantly dysregulated in GDM, it was selected as a potential circulating biomarker for this disease [45]. Additionally, as a stress-related miRNA, *miR-223* negatively regulated the cryopyrin-encoding gene *NLRP3* and subsequently interleukin-1 beta production [46]. In our study, production of the TFs zinc finger E-box binding homeobox 1 (ZEB1) and Forkhead box O1 (FOXO1) was regulated by *miR-223-3p*. FOXO1, a target of insulin signaling, regulates metabolic homeostasis in response to oxidative stress. The interaction of FOXO1 with β-catenin could attenuate the WNT signaling pathway, which is involved in lipid metabolism and glucose homeostasis [47]. Besides this, FOXO1 was targeted by *miR-520 h* and *miR-520 g-3p*, which were speculated to influence insulin sensitivity in human white adipose tissue through their predicted effects on glucose metabolism [48]. ZEB1, a zinc finger TF, is associated with placental development. It was reported that ZEB1 cooperated with FOXO members to suppress B-lymphocyte proliferation [49]. TBP is a universal eukaryotic TF. It was found that the enhancement of TBP-2 expression caused impairment of glucose-induced insulin secretion and insulin sensitivity [50]. In the present study, TBP was found to regulate many *HLA* genes (*HLA-DQA1*, *HLA-F*, and *HLA-DQA2*), implying its indispensable role in GDM.

## Conclusions

In conclusion, four immune-related DEGs of GDM (viz., *HLA*, *CXCL9*, *CXCL10*, and *PRPTC*) appeared to be associated with not only the autoimmune process but also residual β-cell function. *miR-223-3p*, *miR-520* (i.e., *miR-520 h* and *miR-520 g-3p*), and TBP regulated most of the DEGs, especially cellular metabolism-related genes (*FOXO1* and *ZEB1*). These results provide new insights into the mechanisms of GDM pathogenesis.

## Data Availability

All data generated or analyzed during this study are included in this published article.

## Abbreviations

BP: Biological process
CC: Cellular component
CYP1A1: Cytochrome P450, family 1, subfamily A, polypeptide 1
DEGs: Differentially expressed genes
FOXO1: Forkhead box O1
GDM: Gestational diabetes mellitus
GO: Gene Ontology
KEGG: Kyoto Encyclopedia of Genes and Genomes
MF: Molecular function
POU1F1: POU class 1 homeobox 1
PPI: Protein-protein interaction
PTP1B: Protein tyrosine phosphatase 1B
PTPRC: Protein tyrosine phosphatase, receptor type C
TBP: Thioredoxin-binding protein
TF: Transcription factor
ZEB1: Zinc finger E-box-binding homeobox 1
